# Efficacy of tezepelumab in patients with severe asthma and persistent airflow obstruction

**DOI:** 10.1183/23120541.00164-2024

**Published:** 2024-11-25

**Authors:** Elliot Israel, Mario Castro, Christopher S. Ambrose, Jean-Pierre Llanos, Nestor A. Molfino, Nicole L. Martin, Sandhia S. Ponnarambil, Neil Martin

**Affiliations:** 1 Pulmonary and Critical Care Medicine, Allergy and Immunology, Brigham and Women's Hospital, Harvard Medical School, Boston, MA, USA; 2 Division of Pulmonary, Critical Care and Sleep Medicine, University of Kansas School of Medicine, Kansas City, KS, USA; 3 Respiratory and Immunology, BioPharmaceuticals Medical, AstraZeneca, Gaithersburg, MD, USA; 4 Global Medical Affairs, Amgen, Thousand Oaks, CA, USA; 5 Global Development, Amgen, Thousand Oaks, CA, USA; 6 Biometrics, Late-stage Development, Respiratory and Immunology, BioPharmaceuticals R&D, AstraZeneca, Waltham, MA, USA; 7 Cytel Inc., Waltham, MA, USA; 8 Late-stage Development, Respiratory and Immunology, BioPharmaceuticals R&D, AstraZeneca, Cambridge, UK; 9 Respiratory and Immunology, BioPharmaceuticals Medical, AstraZeneca, Cambridge, UK; 10 University of Leicester, Leicester, UK

## Abstract

**Background:**

Persistent airflow obstruction (PAO) in patients with asthma can be difficult to treat. Tezepelumab blocks thymic stromal lymphopoietin, an epithelial cytokine implicated in asthma pathogenesis. This analysis evaluated the efficacy of tezepelumab in patients with severe, uncontrolled asthma and PAO.

**Methods:**

PATHWAY (phase 2b) and NAVIGATOR (phase 3) were multicentre, randomised, double-blind, placebo-controlled studies. This *post hoc* analysis included PATHWAY and NAVIGATOR patients who received tezepelumab 210 mg or placebo every 4 weeks for 52 weeks. Change from baseline to week 52 in pre-bronchodilator forced expiratory volume in 1 s (FEV_1_) and the annualised asthma exacerbation rate (AAER) over 52 weeks were assessed in patients with and without PAO (post-bronchodilator FEV_1_/forced vital capacity ratio <0.7) at baseline.

**Results:**

Of the 1334 included patients, 782 (58.6%) had PAO at baseline. At week 52, greater improvements in pre-bronchodilator FEV_1_ from baseline were observed in tezepelumab *versus* placebo recipients with PAO (least-squares (LS) mean 0.24 *versus* 0.07 L; difference 0.17 L, 95% confidence interval (CI): 0.11–0.23) and without PAO (LS mean 0.20 *versus* 0.12 L; difference 0.08 L, 95% CI: 0.01–0.15). Tezepelumab reduced the AAER *versus* placebo by 61% (95% CI: 51–69) and 56% (95% CI: 42–67) in patients with and without PAO, respectively. For patients with PAO at baseline, the proportion without PAO at week 52 was higher with tezepelumab (12.1%) than placebo (6.6%) (odds ratio 1.96, 95% CI: 1.30–2.94).

**Conclusion:**

Tezepelumab improved lung function and reduced exacerbations *versus* placebo in patients with severe, uncontrolled asthma with and without PAO.

## Shareable abstract

**Treatment with tezepelumab improved lung function and reduced asthma exacerbations compared with placebo in patients with severe, uncontrolled asthma and persistent airflow obstruction, which can be difficult to treat** https://bit.ly/3XoPkPM

## Introduction

Airflow obstruction that is reversible, either spontaneously or following treatment with bronchodilators or corticosteroids, is a common feature of asthma [1, 2]. However, airflow obstruction is not always completely reversible, especially in patients with severe asthma. Some patients continue to experience obstructed airways, known as persistent airflow obstruction (PAO), which is often difficult to treat [3].

PAO has been defined as a predicted post-bronchodilator forced expiratory volume in 1 s (FEV_1_)/forced vital capacity (FVC) ratio of <0.7, per the criteria from the Global Initiative for Chronic Obstructive Lung Disease [4]. PAO is a predictor of overall mortality in patients with asthma [5] and is associated with more severe disease [6, 7]. Chronic airway inflammation and airway remodelling are mechanisms central to asthma pathophysiology [8, 9] and are thought to contribute to the development of PAO [9, 10].

Tezepelumab is a human monoclonal antibody that blocks thymic stromal lymphopoietin (TSLP) [11, 12], an epithelial cytokine implicated in multiple aspects of asthma pathophysiology [13]. The US Food and Drug Administration and the European Commission have approved tezepelumab for the treatment of severe asthma with no phenotype or biomarker limitations [14, 15]. In the phase 2b PATHWAY (ClinicalTrials.gov identifier: NCT02054130) and phase 3 NAVIGATOR (ClinicalTrials.gov identifier: NCT03347279) studies, tezepelumab significantly reduced the annualised asthma exacerbation rate (AAER) and improved lung function and asthma control compared with placebo in a broad population of patients with severe, uncontrolled asthma across different phenotypes and baseline biomarker levels [11, 12, 16].

In the phase 2 CASCADE study (ClinicalTrials.gov identifier: NCT03688074), tezepelumab treatment led to a reduction in airway hyperresponsiveness to mannitol compared with placebo [17], which was also observed in the phase 2 UPSTREAM study (ClinicalTrials.gov identifier: NCT02698501) [18]. Given that smooth muscle pathology and airway inflammation are thought to contribute to both airway hyperresponsiveness and PAO in asthma [9, 10, 19], tezepelumab may have a reductive effect on PAO in patients with asthma. Additionally, TSLP can promote airway remodelling through activation of human lung fibroblasts [20]; recent evidence suggests that fibroblasts represent both a source and target of TSLP [21, 22], which may implicate TSLP as a therapeutic target for airway remodelling in asthma.

Airway mucus plugging is frequently found in patients with severe asthma and has also been shown to contribute to the development of PAO [23]. In a recent analysis of CASCADE, tezepelumab treatment was associated with a reduction compared with placebo in occlusive mucus plugs in the airways of patients with asthma [24]. The reduction in mucus plugs observed was also associated with improvement in lung function [24]. These findings, together with studies demonstrating the effect of mucus plugs on distal airflow [25, 26], identify another possible mechanism through which tezepelumab treatment may lead to a reduction in PAO in asthma.

This *post hoc* analysis evaluated the efficacy of tezepelumab in a pooled population of patients with severe, uncontrolled asthma and PAO from PATHWAY and NAVIGATOR. This is the first analysis to assess the efficacy of tezepelumab in patients with PAO.

## Study design and methods

PATHWAY and NAVIGATOR were multicentre, randomised, double-blind, placebo-controlled, parallel-group, 52-week studies with similar designs and eligibility criteria, conducted in patients with severe, uncontrolled asthma (supplementary figure S1) [11, 12]. PATHWAY was a phase 2b study conducted between December 2013 and March 2017 [12]. NAVIGATOR was a phase 3 study conducted between November 2017 and September 2020 [11]. The full study design details for PATHWAY and NAVIGATOR have been described previously [11, 12, 27].

Both studies were conducted in accordance with the ethical principles of the Declaration of Helsinki, the International Council for Harmonisation good clinical practice guidelines and applicable regulatory requirements. Approvals from independent ethics committees were obtained, and all patients or their guardians provided written informed consent in accordance with local requirements.

### Patients

Patients in PATHWAY and NAVIGATOR (18–75 years old and 12–80 years old, respectively) were nonsmokers at the time of the studies, with physician-diagnosed asthma and a post-bronchodilator FEV_1_ reversibility of at least 12% and at least 200 mL during screening or during the 12 months before screening. Patients were also required to have a history of at least two asthma exacerbations (or, in the case of PATHWAY, at least one severe exacerbation that led to hospitalisation) in the 12 months before the date of informed consent. Exacerbations were defined as a worsening of asthma symptoms that led to hospitalisation, an emergency room visit resulting in the use of systemic corticosteroids for at least 3 consecutive days or the use of systemic corticosteroids for at least 3 consecutive days).

In PATHWAY, patients had to have documented physician-diagnosed asthma for at least 12 months before visit 1, which was uncontrolled despite receiving treatment with medium- or high-dose inhaled corticosteroids (ICS) (fluticasone propionate 250–500 µg·day^−1^ or >500 µg·day^−1^ or equivalent, respectively) [2] plus a long-acting β_2_ agonist for at least 6 months before enrolment, with or without oral corticosteroids (OCS). In NAVIGATOR, patients had to have been receiving medium- or high-dose ICS (fluticasone propionate ≥500 µg·day^−1^ or equivalent) for at least 12 months before screening and at least one additional controller medication, with or without OCS, for at least 3 months before the date of informed consent.

### Procedures

In PATHWAY, eligible patients were randomised (1:1:1:1) to receive either tezepelumab 70 mg every 4 weeks (Q4W), 210 mg Q4W or 280 mg every 2 weeks (Q2W) or placebo Q2W subcutaneously over 52 weeks. The treatment period was followed by a 12-week post-treatment follow-up period. In NAVIGATOR, patients were randomised (1:1) to receive tezepelumab 210 mg Q4W or placebo subcutaneously over 52 weeks. At week 52, patients entered a 12-week post-treatment follow-up period or the DESTINATION long-term extension study (ClinicalTrials.gov identifier: NCT03706079).

### End-points

All end-points in this analysis were assessed for the pooled patient population of the PATHWAY (tezepelumab 210 mg Q4W and placebo groups only) and NAVIGATOR studies. PAO was defined as a post-bronchodilator FEV_1_/FVC ratio of <0.7 at baseline [4].

Changes from baseline to week 52 in pre-bronchodilator FEV_1_ (minimum clinically important difference, 0.1 L) [28], post-bronchodilator FEV_1_, pre-bronchodilator FVC, post-bronchodilator FVC, post-bronchodilator FEV_1_/FVC ratio and post-bronchodilator FEV_1_ % reversibility were assessed by treatment group and PAO subgroup (patients with or without PAO at baseline). The change from baseline to week 52 in pre-bronchodilator FEV_1_ was also assessed in patients grouped by PAO subgroup and the following baseline biomarker levels: blood eosinophil count (BEC; <150, ≥150, <300 and ≥300 cells·µL^−1^) and fractional exhaled nitric oxide (*F*_ENO_) levels (<25, ≥25, <50 and ≥50 ppb).

The AAER over 52 weeks was assessed in patients with and without PAO at baseline and in patients grouped by PAO subgroup and baseline biomarker level (BEC and *F*_ENO_; biomarker cut-offs were the same as those for the pre-bronchodilator FEV_1_ analysis). To assess the relationship between the magnitude of airway obstruction and the clinical burden of asthma, the AAER over 52 weeks was also assessed in patients grouped by post-bronchodilator FEV_1_/FVC ratio at baseline (<50%, 50 to <60%, 60 to <70% and ≥70%). The proportions of patients transitioning between PAO subgroups (patients with or without PAO) from baseline to the end of treatment were assessed by treatment group. The proportions of patients transitioning between % predicted pre-bronchodilator FEV_1_ subgroups (<80% (abnormal lung function) and ≥80% (normal lung function)) from baseline to the end of treatment were also assessed by PAO subgroup.

### Statistical analysis

Changes from baseline to week 52 in pre-bronchodilator FEV_1_, post-bronchodilator FEV_1_, pre-bronchodilator FVC, post-bronchodilator FVC, post-bronchodilator FEV_1_/FVC ratio and post-bronchodilator FEV_1_ % reversibility were estimated using a repeated measures model. Treatment group, study (PATHWAY or NAVIGATOR), baseline measurement for the relevant lung function parameter, visit, subgroup, treatment-by-visit, treatment-by-subgroup, visit-by-subgroup and treatment-by-visit-by-subgroup were included as covariates. The baseline measurement for the relevant lung function parameter was included as a continuous linear covariate.

The AAER over 52 weeks was estimated using a negative binomial regression model with treatment group, study (PATHWAY or NAVIGATOR), history of exacerbations (≤2 or >2 in the previous 12 months), subgroup and treatment-by-subgroup interaction included as covariates. Data on the proportion of patients transitioning between PAO subgroups and abnormal/normal lung function subgroups are reported descriptively (n, %).

A sensitivity analysis was also conducted in patients with or without PAO at screening/run-in and baseline (both visits) to assess changes in pre- and post-bronchodilator FEV_1_ from baseline to week 52 and the AAER over 52 weeks, as well as the proportion of patients with PAO at screening/run-in and baseline who no longer had PAO at week 52.

## Results

### Baseline demographics and clinical characteristics

Of the 1334 included patients, 782 (58.6%) had PAO at baseline (tezepelumab, n=388; placebo, n=394) and 552 did not (tezepelumab, n=277; placebo, n=275) (table 1).

**TABLE 1:** Baseline demographics and clinical characteristics in patients with and without PAO

	Patients with PAO			Patients without PAO		
Demographic/characteristic	Tezepelumab 210 mg Q4W	Placebo	Overall	Tezepelumab 210 mg Q4W	Placebo	Overall
**Patients, n**	388	394	782	277	275	552
**Age years, mean±sd**	54.5±12.7	53.8±12.2	54.1±12.4	44.9±17.6	43.9±17.0	44.4±17.3
**Female, n (%)**	243 (62.6)	234 (59.4)	477 (61.0)	179 (64.6)	197 (71.6)	376 (68.1)
**Former smoker, n (%)**	88 (22.7)	89 (22.6)	177 (22.6)	45 (16.2)	37 (13.5)	82 (14.9)
**Pack-years among former smokers, mean±sd**	4.3±3.0	4.6±3.3	4.5±3.1	3.6±2.9	3.9±2.9	3.8±2.9
**Age at asthma onset years, n (%)**						
** **<18	121 (31.2)	116 (29.4)	237 (30.3)	106 (38.3)	107 (38.9)	213 (38.6)
** **18–40	140 (36.1)	148 (37.6)	288 (36.8)	83 (30.0)	95 (34.5)	178 (32.2)
** **>40	127 (32.7)	130 (33.0)	257 (32.9)	88 (31.8)	73 (26.5)	161 (29.2)
**Time since asthma diagnosis years, mean±sd**	24.5±16.9	23.1±16.0	23.8±16.5	16.9±13.3	18.1±13.7	17.5±13.5
**BMI kg·m^−2^, mean±sd**	28.5±6.6	28.3±6.1	28.4±6.4	28.9±6.8	28.4±7.3	28.6±7.1
**ICS dose, n (%)^#^**						
** **Medium	97 (25.0)	111 (28.2)	208 (26.6)	104 (37.5)	94 (34.2)	198 (35.9)
** **High	291 (75.0)	282 (71.6)	573 (73.3)	173 (62.5)	181 (65.8)	354 (64.1)
**Additional maintenance treatments (in addition to ICS), n (%)**						
** **LABA	215 (55.4)	215 (54.6)	430 (55.0)	144 (52.0)	149 (54.2)	293 (53.1)
** **LABA+LAMA	44 (11.3)	41 (10.4)	85 (10.9)	21 (7.6)	27 (9.8)	48 (8.7)
** **LABA+LAMA+LTRA	47 (12.1)	39 (9.9)	86 (11.0)	29 (10.5)	23 (8.4)	52 (9.4)
** **LABA+LTRA	79 (20.4)	93 (23.6)	172 (22.0)	80 (28.9)	73 (26.5)	153 (27.7)
**OCS use, n (%)**	40 (10.3)	45 (11.4)	85 (10.9)	18 (6.5)	19 (6.9)	37 (6.7)
**Number of exacerbations in the past 12 months, per patient**						
Mean±sd	2.8±1.5	2.8±1.5	2.8±1.5	2.6±1.2	2.5±1.0	2.6±1.1
** **Median (min, max)	2 (1, 15)	2 (1, 11)	2 (1, 15)	2 (1, 10)	2 (1, 8)	2 (1, 10)
**Pre-bronchodilator FEV_1_ L, mean±sd**	1.55±0.53	1.58±0.55	1.57±0.54	2.23±0.70	2.22±0.68	2.22±0.69
**Post-bronchodilator FEV_1_ L, mean±sd**	1.79±0.61	1.84±0.63	1.81±0.62	2.53±0.77	2.54±0.71	2.53±0.74
**% predicted pre-bronchodilator FEV_1_, mean±sd**	53.98±13.99	54.32±14.36	54.15±14.17	73.21±14.45	73.39±14.40	73.30±14.42
**Pre-bronchodilator FVC L, mean±sd**	2.84±0.90	2.91±0.96	2.87±0.93	3.01±0.94	3.01±0.85	3.01±0.90
**Post-bronchodilator FVC L, mean±sd**	3.13±0.96	3.20±1.01	3.16±0.98	3.21±0.96	3.22±0.85	3.21±0.91
**Pre-bronchodilator FEV_1_/FVC %, mean±sd**	54.84±9.45	54.90±9.02	54.87±9.23	74.32±8.34	73.85±8.30	74.09±8.32
**Post-bronchodilator FEV_1_/FVC %, mean±sd**	57.48±9.28	57.50±8.91	57.49±9.09	79.07±6.66	78.74±6.85	78.91±6.75
**FEV_1_ % reversibility, mean±sd**	17.0±16.0	17.1±16.5	17.1±16.3	15.1±17.1	15.9±16.1	15.5±16.6
**FEV_1_ % reversibility, n (%)**						
** **<12	167 (43.0)	173 (43.9)	340 (43.5)	151 (54.5)	142 (51.6)	293 (53.1)
** **≥12 to <15	30 (7.7)	40 (10.2)	70 (9.0)	26 (9.4)	21 (7.6)	47 (8.5)
** **≥15	191 (49.2)	181 (45.9)	372 (47.6)	100 (36.1)	112 (40.7)	212 (38.4)
**Serum total IgE IU·mL^−1^, median (min, max)**	192 (2, 12 823)	180 (2, 11 860)	185 (2, 12 823)	167 (2, 3665)	187 (2, 9741)	174 (2, 9741)
**FEIA positive for any perennial aeroallergen, n (%)^¶^**	223 (57.5)	231 (58.6)	454 (58.1)	187 (67.5)	174 (63.3)	361 (65.4)
**BEC cells·µL^−1^, median (IQR)**	280 (170–470)	280 (150–480)	280 (160–480)	220 (120–380)	230 (130–390)	220 (130–380)
***F***_**ENO**_ **ppb, median (min, max)**	29.0 (4.0, 198.0)	29.0 (5.0, 276.3)	29.0 (4.0, 276.3)	28.0 (5.0, 235.0)	24.0 (3.5, 265.0)	26.5 (3.5, 265.0)

PAO: persistent airflow obstruction; Q4W: every 4 weeks; BMI: body mass index; ICS: inhaled corticosteroid; LABA: long-acting β-agonist; LAMA: long-acting muscarinic antagonist; LTRA: leukotriene receptor antagonist; OCS: oral corticosteroid; FEV_1_: forced expiratory volume in 1 s; FVC: forced vital capacity; IgE: immunoglobulin E; FEIA: fluorescence enzyme immunoassay; BEC: blood eosinophil count; *F*_ENO_: fractional exhaled nitric oxide. ^#^: medium-dose ICS: fluticasone propionate 250–500 μg·day^−1^ or equivalent; high-dose ICS: fluticasone propionate >500 μg·day^−1^ or equivalent. There was one patient from NAVIGATOR with PAO (placebo group) who received fluticasone propionate <500 µg·day^−1^ or equivalent. ^¶^: positive for at least one perennial aeroallergen (cat dander, dog dander, cockroach, dust mite (*Dermatophagoides farinae* or *D. pteronyssinus*) and mould mix).

At baseline, patients with PAO had a higher mean±sd age (54.1±12.4 years *versus* 44.4±17.3 years, respectively), lower pre-bronchodilator FEV_1_ (1.6±0.5 L *versus* 2.2±0.7 L, respectively) and a lower % predicted pre-bronchodilator FEV_1_ (54.15±14.17% *versus* 73.30±14.42%, respectively) than patients without PAO (table 1). Both subgroups had similar FEV_1_ % reversibility at baseline (table 1). Patients with PAO had a longer mean±sd time since asthma diagnosis than those without PAO (23.8±16.5 years *versus* 17.5±13.5 years, respectively; table 1). A larger proportion of patients with PAO compared with those without PAO were former smokers (22.6% *versus* 14.9%, respectively), were receiving high-dose ICS (73.3% *versus* 64.1%, respectively) and were receiving daily OCS (10.9% *versus* 6.7%, respectively; table 1). Patients with PAO had a higher median (interquartile range (IQR)) baseline BEC than those without PAO (280 (160–480) cells·µL^−1^ *versus* 220 (130–380) cells·µL^−1^, respectively; table 1). Conversely, a lower proportion of patients with PAO were sensitised to perennial aeroallergens than those without PAO (58.1% *versus* 65.4%, respectively; table 1). Baseline demographics and clinical characteristics for patients with and without PAO grouped by baseline BEC and *F*_ENO_ are shown in supplementary tables S1 and S2.

### Lung function

**FIGURE 1:**
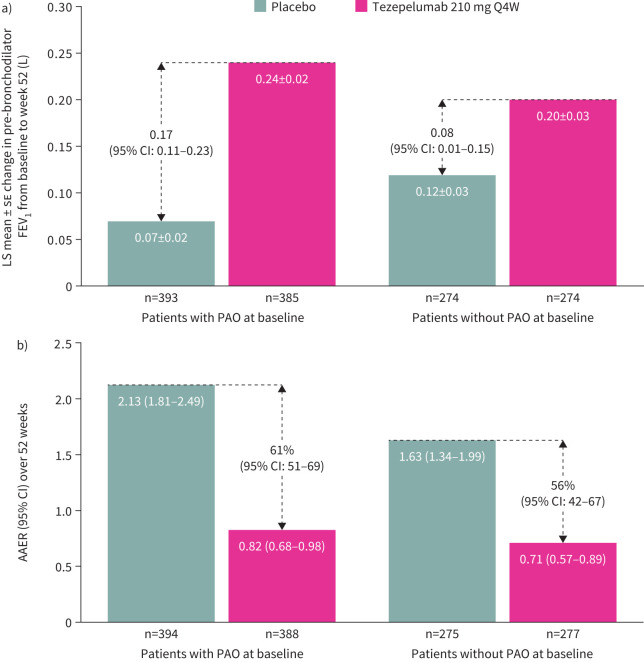
a) Change from baseline to week 52 in pre-bronchodilator FEV_1_ and b) AAER over 52 weeks in patients with and without PAO at baseline. n indicates the number of patients who contributed to the analysis. AAER: annualised asthma exacerbation rate; FEV_1_: forced expiratory volume in 1 s; LS: least-squares; PAO: persistent airflow obstruction; Q4W: every 4 weeks.

Tezepelumab treatment improved pre-bronchodilator FEV_1_ at week 52 compared with placebo: the least-squares (LS) mean±se change in pre-bronchodilator FEV_1_ from baseline to week 52 was 0.24±0.02 L for tezepelumab and 0.07±0.02 L for placebo in patients with PAO (LS mean difference 0.17 L, 95% confidence interval (CI): 0.11–0.23; figure 1a). Improvements from baseline to week 52 were also observed with tezepelumab compared with placebo in post-bronchodilator FEV_1_, pre-bronchodilator FVC, post-bronchodilator FVC and post-bronchodilator FEV_1_/FVC ratio and were numerically greater in patients with PAO at baseline than in those without PAO at baseline (figure 1a and table 2). Improvements in pre- and post-bronchodilator FEV_1_ were similar with tezepelumab *versus* placebo when PAO was present at baseline compared with when PAO was present at baseline and screening/run-in (supplementary table S3). Compared with placebo, treatment with tezepelumab improved pre-bronchodilator FEV_1_ from baseline to week 52 in patients with baseline BECs of ≥150 cells·µL^−1^ or ≥300 cells·µL^−1^ and baseline *F*_ENO_ levels of ≥25 ppb regardless of PAO status (table 3).

**TABLE 2:** Change in lung function parameters from baseline to week 52 in patients with and without PAO

	Tezepelumab 210 mg Q4W			Placebo			LS mean difference (95% CI)
	Baseline, mean±sd	n	LS mean±se change from baseline	Baseline, mean±sd	n	LS mean±se change from baseline	
**Patients with PAO**							
** **Pre-bronchodilator FVC L	2.84±0.90	385	0.25±0.02	2.91±0.96	393	0.08±0.02	0.17 (0.10–0.23)
** **Post-bronchodilator FVC L	3.13±0.96	356	0.13±0.02	3.20±1.01	348	0.00±0.02	0.13 (0.07–0.19)
** **Post-bronchodilator FEV_1_ L	1.79±0.61	356	0.18±0.02	1.84±0.63	348	0.02±0.02	0.15 (0.10–0.21)
** **Post-bronchodilator FEV_1_/FVC %	57.48±9.28	356	2.09±0.40	57.50±8.91	348	−0.38±0.40	2.47 (1.51–3.43)
** **Post-bronchodilator FEV_1_ reversibility %	17.00±16.00	352	−5.68±0.62	17.11±16.54	344	−3.33±0.63	−2.35 (−4.08–−0.62)
**Patients without PAO**							
** **Pre-bronchodilator FVC L	3.01±0.94	274	0.20±0.03	3.01±0.85	274	0.11±0.03	0.09 (0.01–0.17)
** **Post-bronchodilator FVC L	3.21±0.96	245	0.10±0.03	3.22±0.85	242	0.02±0.03	0.08 (0.01–0.15)
** **Post-bronchodilator FEV_1_ L	2.53±0.77	245	0.07±0.03	2.54±0.71	242	−0.01±0.03	0.08 (0.01–0.14)
** **Post-bronchodilator FEV_1_/FVC %	79.07±6.66	245	0.91±0.51	78.74±6.85	242	0.67±0.51	0.24 (−0.92–1.39)
** **Post-bronchodilator FEV_1_ reversibility %	15.14±17.12	243	−7.62±0.75	15.87±16.11	240	−6.66±0.75	−0.97 (−3.04–1.11)

PAO: persistent airflow obstruction; Q4W: every 4 weeks; LS: least-squares; FVC: forced vital capacity; FEV_1_: forced expiratory volume in 1 s.

**TABLE 3:** Change in pre-bronchodilator FEV_1_ from baseline to week 52 in patients with and without PAO by baseline BEC and *F*_ENO_ level

Biomarker level at baseline	Patients with PAO					Patients without PAO				
	LS mean±se change from baseline L				LS mean difference (95% CI) L	LS mean±se change from baseline L				LS mean difference (95% CI) L
	Tezepelumab 210 mg Q4W	n	Placebo	n		Tezepelumab 210 mg Q4W	n	Placebo	n	
**BEC cells·µL^−1^**										
** **<150	0.03±0.05	79	−0.01±0.04	91	0.04 (−0.08–0.17)	0.10±0.05	87	0.17±0.05	80	−0.07 (−0.19–0.06)
** **≥150	0.30±0.02	306	0.10±0.02	302	0.20 (0.14–0.27)	0.24±0.03	187	0.10±0.03	194	0.15 (0.07–0.23)
** **<300	0.10±0.03	198	0.02±0.03	209	0.08 (0.00–0.16)	0.13±0.03	177	0.11±0.03	172	0.03 (−0.06–0.11)
** **≥300	0.40±0.03	187	0.13±0.03	184	0.27 (0.18–0.35)	0.32±0.04	97	0.13±0.04	102	0.19 (0.08–0.30)
***F***_**ENO**_ **level ppb**										
** **<25	0.12±0.03	166	0.04±0.03	157	0.08 (−0.01–0.17)	0.15±0.04	123	0.12±0.04	136	0.03 (−0.07–0.13)
** **≥25	0.33±0.03	217	0.09±0.03	233	0.24 (0.17–0.32)	0.25±0.04	145	0.12±0.04	136	0.14 (0.04–0.23)
** **<50	0.17±0.03	286	0.04±0.03	275	0.13 (0.07–0.20)	0.20±0.03	193	0.11±0.03	198	0.09 (0.01–0.17)
** **≥50	0.44±0.04	97	0.14±0.04	115	0.30 (0.19–0.41)	0.23±0.05	75	0.15±0.05	74	0.08 (−0.05–0.21)

FEV_1_: forced expiratory volume in 1 s; PAO: persistent airflow obstruction; BEC: blood eosinophil count; *F*_ENO_: fractional exhaled nitric oxide; LS: least-squares; Q4W: every 4 weeks.

### Exacerbations

Among placebo recipients, the AAER over 52 weeks was higher in patients with PAO (2.13 (95% CI: 1.81–2.49)) than in those without PAO (1.63 (95% CI: 1.34–1.99); figure 1b). Tezepelumab treatment reduced the AAER over 52 weeks by 61% (95% CI: 51, 69) and 56% (95% CI: 42, 67) compared with placebo in patients with and without PAO, respectively (figure 1b).

**FIGURE 2:**
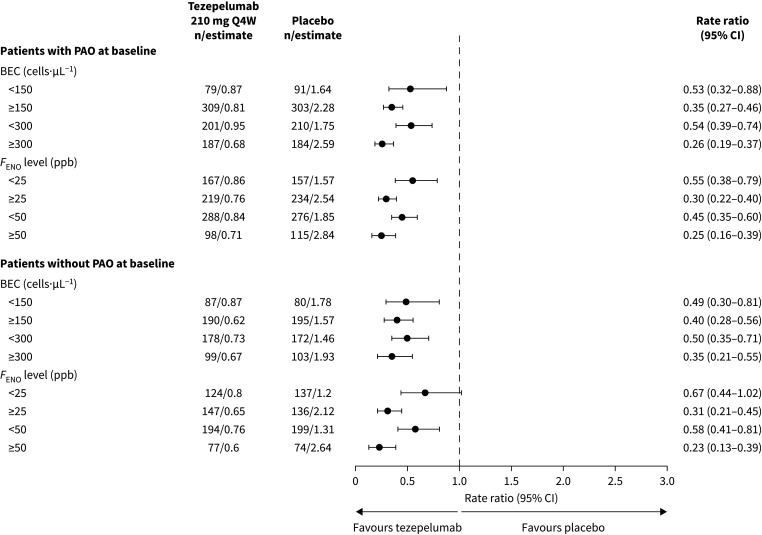
AAER over 52 weeks in patients with and without PAO at baseline by baseline BEC and *F*_ENO_ level. n indicates the number of patients who contributed to the analysis. AAER: annualised asthma exacerbation rate; BEC: blood eosinophil count; *F*_ENO_: fractional exhaled nitric oxide; PAO: persistent airflow obstruction; Q4W: every 4 weeks.

Compared with placebo, tezepelumab treatment led to reductions in the AAER over 52 weeks in patients with and without PAO across BEC and *F*_ENO_ subgroups (figure 2); reductions ranged from 33% to 77%. The greatest reductions were observed in patients with high baseline BECs or high baseline *F*_ENO_ levels, irrespective of PAO status. In patients with a baseline post-bronchodilator FEV_1_/FVC ratio of <50%, 50 to <60% or 60 to <70%, tezepelumab reduced the AAER over 52 weeks compared with placebo by 69% (95% CI: 50–81), 71% (95% CI: 56–80) and 44% (95% CI: 21–60), respectively. Reductions in the AAER over 52 weeks with tezepelumab *versus* placebo were similar when PAO was present at baseline compared with when PAO was present at screening/run-in and baseline (supplementary table S3).

### PAO and lung function subgroup transitions

**FIGURE 3:**
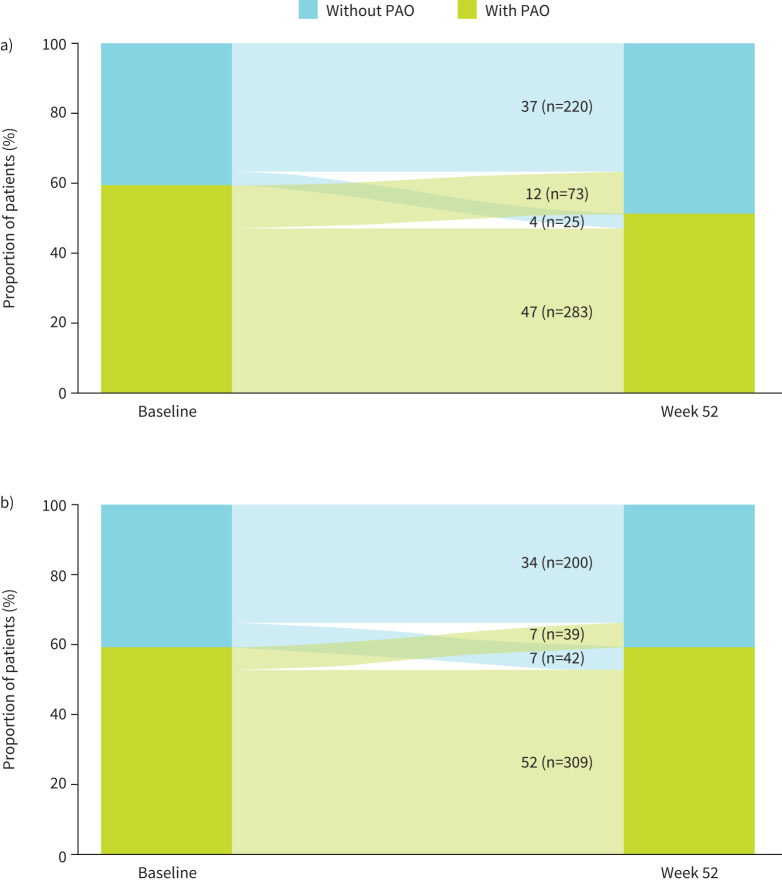
Transition of a) tezepelumab and b) placebo recipients between PAO subgroups from baseline to week 52. PAO: persistent airflow obstruction.

For patients with PAO at baseline, the proportion of patients who did not have PAO at week 52 was higher in the tezepelumab group (12%; n=73 out of 601) than in the placebo group (7%; n=39 out of 590) (odds ratio 1.96, 95% CI: 1.30–2.94) (figure 3). Similar results were observed when PAO was present at baseline compared with when PAO was present at screening/run-in and baseline (supplementary table S3).

Among tezepelumab recipients, patients with PAO at baseline who did not have PAO at week 52 had a lower mean±sd age than those who had PAO at baseline and at week 52 (48.5±14.7 years *versus* 56.2±11.4 years, respectively; table 4) and a shorter mean±sd time since asthma diagnosis (19.1±14.3 years *versus* 26.1±17.5 years, respectively; table 4). Additionally, patients with PAO at baseline who did not have PAO at week 52 had a higher median (IQR) baseline BEC than patients who had PAO at baseline and at week 52 (340 (240–540) cells·µL^−1^ *versus* 260 (160–450) cells·µL^−1^, respectively; table 4), as well as a higher median (min, max) baseline *F*_ENO_ level (45.0 (5.0, 174.0) ppb *versus* 26.0 (4.0, 198.0) ppb, respectively; table 4) and a higher mean post-bronchodilator FEV_1_/FVC ratio at baseline (65% *versus* 56%, respectively; table 4). Baseline demographics and clinical characteristics of placebo recipients who did and did not transition between PAO subgroups are shown in supplementary table S4.

**TABLE 4:** Baseline demographics and clinical characteristics of tezepelumab recipients grouped by whether they had PAO at baseline and/or week 52

Demographic/characteristic	Patients with PAO at baseline and at week 52	Patients without PAO at baseline or at week 52	Patients without PAO at baseline and with PAO at week 52	Patients with PAO at baseline and without PAO at week 52
**Patients, n**	283	220	25	73
**Age years, mean±sd**	56.2±11.4	45.1±17.2	54.2±14.8	48.5±14.7
**Female, n (%)**	171 (60.4)	146 (66.4)	15 (60.0)	51 (69.9)
**Time since asthma diagnosis years, mean±sd**	26.1±17.5	16.5±13.4	21.2±13.7	19.1±14.3
**BMI kg·m^−2^, mean±sd**	28.5±6.8	29.0±6.9	27.1±6.6	28.3±5.4
**ICS dose, n (%)^#^**				
** **Medium	69 (24.4)	87 (39.5)	10 (40.0)	17 (23.3)
** **High	214 (75.6)	133 (60.5)	15 (60.0)	56 (76.7)
**OCS use, n (%)**	28 (9.9)	12 (5.5)	0 (0.0)	7 (9.6)
**Number of exacerbations in the past 12 months, per patient**				
** **Mean±sd	2.8±1.5	2.6±1.1	3.0±1.9	2.8±1.6
** **Median (min, max)	2.0 (1, 15)	2.0 (1, 10)	2.0 (2, 10)	2.0 (2, 13)
**Pre-bronchodilator FEV_1_ L, mean±sd**	1.49±0.50	2.24±0.70	2.01±0.70	1.79±0.56
**Post-bronchodilator FEV_1_ L, mean±sd**	1.73±0.58	2.54±0.75	2.20±0.72	2.06±0.65
**% predicted pre-bronchodilator FEV_1_, mean±sd**	52.65±14.08	73.69±13.42	67.53±15.58	60.15±12.56
**Pre-bronchodilator FVC L, mean±sd**	2.83±0.89	2.99±0.92	2.95±1.05	2.92±0.91
**Post-bronchodilator FVC L, mean±sd**	3.13±0.96	3.20±0.94	2.98±1.04	3.18±0.96
**Pre-bronchodilator FEV_1_/FVC %, mean±sd**	53.07±9.20	75.14±8.01	68.81±8.87	61.56±7.46
**Post-bronchodilator FEV_1_/FVC %, mean±sd**	55.56±9.27	79.53±6.73	74.65±5.69	64.75±5.12
**Reversibility in FEV_1_ L, mean±sd**	58.15±159.49	105.75±295.52	55.82±139.19	57.65±175.73
**FEV_1_ % reversibility, n (%)**				
** **<12	119 (42.0)	119 (54.1)	14 (56.0)	35 (47.9)
** **≥12 to <15	26 (9.2)	23 (10.5)	2 (8.0)	1 (1.4)
** **≥15	138 (48.8)	78 (35.5)	9 (36.0)	37 (50.7)
**Serum total IgE IU·mL^−1^, median (min, max)**	196 (2, 12 823)	142 (2, 3665)	255 (21, 2877)	173 (2, 5115)
**FEIA positive for any perennial aeroallergen, n (%)^¶^**	168 (59.4)	143 (65.0)	18 (72.0)	38 (52.1)
**BEC cells·µL^−1^, median (IQR)**	260 (160–450)	215 (120–375)	200 (120–310)	340 (240–540)
***F***_**ENO**_ **ppb, median (min, max)**	26.0 (4.0, 198.0)	27.0 (5.0, 235.0)	23.0 (8.0, 121.7)	45.0 (5.0, 174.0)

PAO: persistent airflow obstruction; BMI: body mass index; ICS: inhaled corticosteroid; OCS: oral corticosteroid; FEV_1_: forced expiratory volume in 1 s; FVC: forced vital capacity; IgE: immunoglobulin E; FEIA: fluorescence enzyme immunoassay; BEC: blood eosinophil count; *F*_ENO_: fractional exhaled nitric oxide. ^#^: medium-dose ICS: fluticasone propionate 250–500 μg·day^−1^ or equivalent; high-dose ICS: fluticasone propionate >500 μg·day^−1^ or equivalent. There was one patient from NAVIGATOR with PAO (placebo group) who received fluticasone propionate <500 µg·day^−1^ or equivalent. ^¶^: positive for at least one perennial aeroallergen (cat dander, dog dander, cockroach, dust mite (*Dermatophagoides farinae* or *D. pteronyssinus*) and mould mix).

**FIGURE 4:**
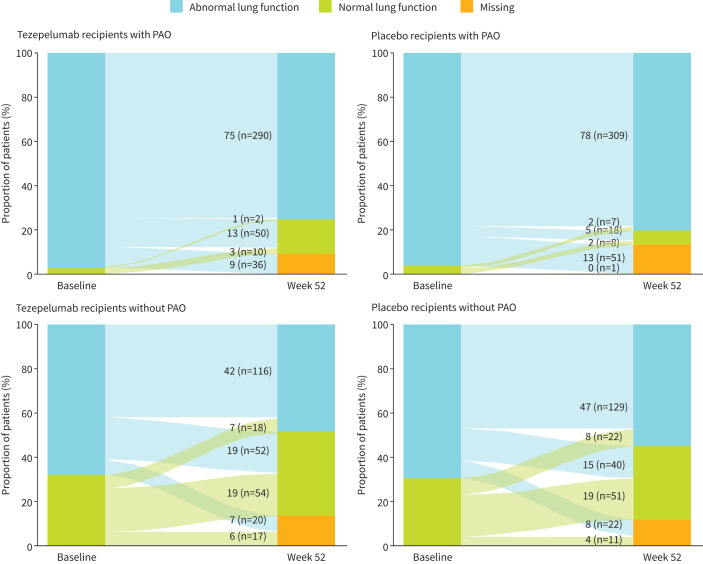
Transition of patients with PAO and without PAO between normal and abnormal lung function subgroups from baseline to week 52. Normal lung function: pre-bronchodilator FEV_1_ ≥80% predicted; abnormal lung function: pre-bronchodilator FEV_1_ <80% predicted. FEV_1_: forced expiratory volume in 1 s; PAO: persistent airflow obstruction.

In patients with PAO, 97% (n=376 out of 388) of tezepelumab recipients and 96% (n=378 out of 394) of placebo recipients had abnormal lung function (% predicted pre-bronchodilator FEV_1_ <80%) at baseline. At week 52, 75% (n=292 out of 388) of tezepelumab recipients and 80% (n=316 out of 394) of placebo recipients with PAO had abnormal lung function (figure 4).

## Discussion

Asthma with PAO is a clinical phenotype defined by a reduced post-bronchodilator FEV_1_/FVC ratio and is associated with accelerated lung function decline and increased risk of morbidity [3]. In this *post hoc* analysis of patients from the PATHWAY and NAVIGATOR studies, tezepelumab treatment improved lung function and reduced asthma exacerbations compared with placebo in patients with severe, uncontrolled asthma with and without PAO.

In the overall population, improvements in lung function compared with placebo were greater in patients with PAO than in those without PAO. These differences were greatest in patients with high type 2 inflammation at baseline, as indicated by high BECs or *F*_ENO_ levels. The observed improvements in post-bronchodilator FEV_1_ and FVC suggest that tezepelumab treatment has additional benefits beyond those achieved by the effects of β-agonists on bronchial smooth muscle.

PAO in asthma is characterised by increased airway smooth muscle, fibrosis and goblet cell hyperplasia and is thought to occur through progressive airway remodelling [29, 30]. Airway inflammation is also thought to contribute to airway remodelling and, therefore, the development of PAO in asthma [9, 10]. While the mechanism of action of tezepelumab that results in improved airway function is unclear, it may be related to the multiple pathways through which TSLP has been shown to drive airway inflammation and remodelling [13, 20]. In addition to driving type 2 and non-type 2 inflammation [31], TSLP expression is increased in airway smooth muscle in asthma and may play a role in the interactions between airway smooth muscle and mast cells [32].

Asthma exacerbation rates were higher among patients who received placebo with PAO than those without PAO. However, tezepelumab treatment reduced asthma exacerbation rates to a similar level in those with and without PAO. Consistent with the overall findings of the NAVIGATOR study [11], the greatest reductions in exacerbations were observed in patients with high baseline inflammatory biomarker levels (BECs ≥300 cells·µL^−1^ and *F*_ENO_ levels ≥25 ppb). Furthermore, clinically meaningful improvements in pre-bronchodilator FEV_1_ were observed with tezepelumab compared with placebo in patients with high inflammatory biomarker levels, irrespective of whether they had PAO. Greater reductions in exacerbations over 52 weeks with tezepelumab compared with placebo were observed in patients with more severe PAO (post-bronchodilator FEV_1_/FVC ratio of <50% and 50% to <60%) than in those with less severe PAO (post-bronchodilator FEV_1_/FVC ratio of 60% to <70% and ≥70%).

Despite the observed improvements in clinical outcomes in patients with PAO at baseline, very few of these patients transitioned to no longer having PAO at week 52. Those who did make this transition were on average younger and had a shorter disease duration. This suggests that initiation of tezepelumab early in the course of the disease may be important in preventing permanent airway remodelling. Additionally, optimisation of maintenance therapy, ensuring adherence to maintenance treatment and the cost-effectiveness of early biologic initiation would need to be considered.

There is a significant clinical overlap between patients with severe asthma and PAO and those with a spirometric diagnosis of COPD [33]; however, the data from this analysis demonstrate that patients with severe asthma and PAO, in particular, exhibit a response to tezepelumab treatment. Tezepelumab is currently undergoing evaluation for patients with clearly defined diagnostic criteria for COPD in a phase 2 clinical trial (COURSE; ClinicalTrials.gov identifier: NCT04039113).

While there is an absence of a standard definition of PAO in asthma [34], the most commonly used definition of PAO (post-bronchodilator FEV_1_/FVC ratio of <0.7 at baseline) was used in this analysis [4], which aligns with studies of other biologics for severe asthma [35–37]. A sensitivity analysis was also completed to assess the efficacy of tezepelumab in patients with or without PAO according to the above criteria but at two time points 4 weeks apart (screening/run-in and baseline). Results from this sensitivity analysis were similar to those when PAO was present at baseline. Of note, it has been estimated that around a third of patients who meet the above definition of PAO still have a positive bronchodilator response [37]. Furthermore, owing to age-related differences in lung function, the fixed post-bronchodilator FEV_1_/FVC ratio threshold of 0.7 can result in an underestimation of disease in younger patients and an overestimation of disease in older patients [38]. Finally, this analysis was exploratory; the study was not powered to evaluate the impact of tezepelumab treatment in patients with and without PAO. Therefore, the findings should be interpreted as descriptive only.

Tezepelumab treatment improved lung function, including post-bronchodilator lung function, and reduced asthma exacerbations compared with placebo in patients with severe, uncontrolled asthma, and PAO, which can be difficult to treat. These improvements further support the efficacy of tezepelumab across a broad population of patients with severe asthma and suggest possible effects on airway remodelling.

## Supplementary material

10.1183/23120541.00164-2024.Supp1 **Please note:** supplementary material is not edited by the Editorial Office, and is uploaded as it has been supplied by the author.
Supplementary material 00164-2024.SUPPLEMENT

## Data Availability

Data underlying the findings described in this article may be obtained in accordance with AstraZeneca's data sharing policy described at https://astrazenecagrouptrials.pharmacm.com/ST/Submission/Disclosure.
