# Supplementary material for: Efficacy of tezepelumab in patients with severe asthma and persistent airflow obstruction
Source: ERJ Open Res. 2024 Nov 25;10(6):00164-2024. doi: 10.1183/23120541.00164-2024 (PMC11587167; doi:10.1183/23120541.00164-2024)
Supplement: Supplementary file 1 [file 00164-2024.SUPPLEMENT.pdf]

Efficacy of tezepelumab in patients with severe asthma and persistent airflow obstruction

Supplementary material

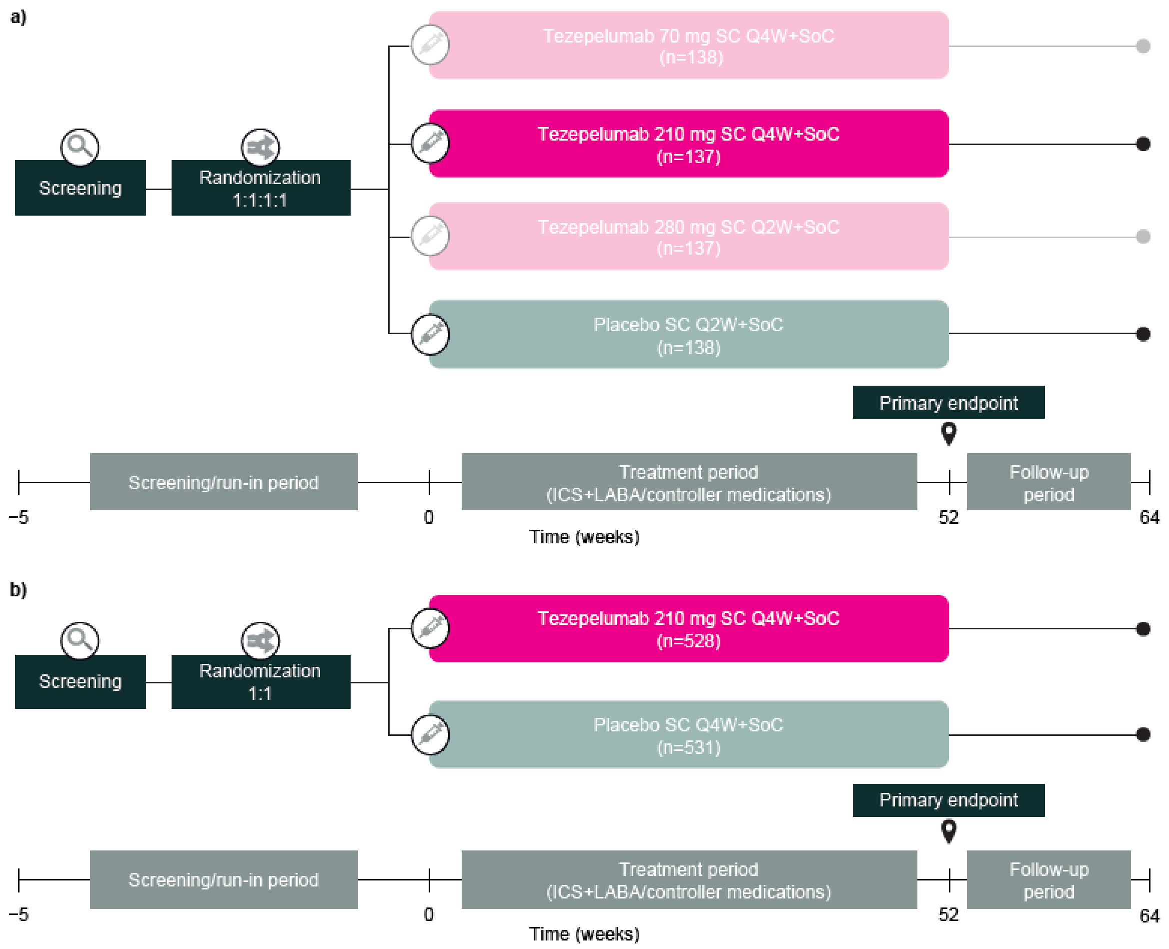

SUPPLEMENTARY FIGURE S1 PATHWAY A) and NAVIGATOR B) study designs.

ICS: inhaled corticosteroid; LABA: long-acting β2 agonist; Q2W: every 2 weeks; Q4W: every 4 weeks; SC: subcutaneously; SoC: standard of care.

SUPPLEMENTARY TABLE S1 Baseline demographics and clinical characteristics in patients with and without PAO at baseline by baseline BEC

	Patients with PAO		Patients without PAO	
Demographic/characteristic	BEC <300 cells·µL^{-1} (n=411)	BEC ≥300 cells·µL^{-1} (n=371)	BEC <300 cells·µL^{-1} (n=350)	BEC ≥300 cells·µL^{-1} (n=202)
Age, years, mean (SD)	55.7 (11.9)	52.4 (12.8)	45.8 (16.6)	41.9 (18.3)
Female, n (%)	246 (59.9)	231 (62.3)	244 (69.7)	132 (65.3)
Age at asthma onset, years, n (%)				
<18	131 (31.9)	106 (28.6)	127 (36.3)	86 (42.6)
18–40	144 (35.0)	144 (38.8)	116 (33.1)	62 (30.7)
>40	136 (33.1)	121 (32.6)	107 (30.6)	54 (26.7)
Time since asthma diagnosis, years, mean (SD)	25.4 (17.3)	22.0 (15.3)	18.3 (13.5)	16.1 (13.4)
BMI, kg·m^{-2}, mean (SD)	28.9 (6.8)	27.9 (5.8)	29.0 (7.1)	28.0 (7.1)
ICS dose, n (%)[#]				
Medium	115 (28.0)	93 (25.1)	134 (38.3)	64 (31.7)
High	295 (71.8)	278 (74.9)	216 (61.7)	138 (68.3)
OCS use, n (%)	48 (11.7)	37 (10.0)	24 (6.9)	13 (6.4)
Number of exacerbations in the past 12 months, per patient				

Mean (SD)	2.7 (1.3)	3.0 (1.7)	2.5 (1.1)	2.7 (1.2)
Median (min, max)	2 (1, 12)	2 (1, 15)	2 (1, 10)	2 (1, 10)
Pre-bronchodilator FEV_1, L, mean (SD)	1.60 (0.56)	1.53 (0.52)	2.19 (0.66)	2.28 (0.74)
Post-bronchodilator FEV_1, L, mean (SD)	1.83 (0.63)	1.79 (0.61)	2.49 (0.72)	2.62 (0.77)
Percent predicted pre-bronchodilator FEV_1, mean (SD)	55.87 (14.10)	52.25 (14.02)	73.27 (13.35)	73.34 (16.13)
Pre-bronchodilator FVC, L, mean (SD)	2.89 (0.94)	2.86 (0.92)	2.97 (0.87)	3.08 (0.94)
Post-bronchodilator FVC, L, mean (SD)	3.17 (0.97)	3.16 (1.00)	3.16 (0.88)	3.31 (0.94)
Pre-bronchodilator FEV_1/FVC %, mean (SD)	55.61 (9.14)	54.05 (9.27)	74.01 (8.27)	74.22 (8.42)
Post-bronchodilator FEV_1/FVC %, mean (SD)	57.93 (9.16)	57.00 (9.00)	78.74 (6.63)	79.19 (6.97)
FEV_1 reversibility, L, mean (SD)	50.9 (149.1)	79.2 (203.2)	90.8 (264.8)	114.1 (305.7)
FEV_1 reversibility, %, mean (SD)	15.83 (14.59)	18.42 (17.83)	14.87 (16.12)	16.59 (17.41)
Serum total IgE, IU/mL, median (min, max)	125 (2, 12 823)	228 (2, 11 430)	139 (2, 5995)	260 (3, 9741)

BEC, cells·μL^{-1}, median (IQR)	170 (100–230)	490 (370–710)	150 (100–220)	470 (370–640)
FeNO, ppb, median (min, max)	24.0 (4.0, 210.0)	38.0 (5.0, 276.3)	20.3 (5.0, 216.0)	45.0 (3.5, 265.0)

BEC: blood eosinophil count; BMI: body mass index; FeNO: fractional exhaled nitric oxide; FEV$_1$: forced expiratory volume in 1 second; FVC: forced vital capacity; ICS: inhaled corticosteroid; IgE: immunoglobulin E; IQR: interquartile range; OCS: oral corticosteroid; PAO: persistent airflow obstruction; SD: standard deviation.

[#]Medium-dose ICS: fluticasone propionate 250–500 μg/day or equivalent; high-dose ICS: fluticasone propionate >500 μg/day or equivalent. There was one patient in NAVIGATOR with PAO and a BEC <300 cells·μL^{-1} (placebo group) who received fluticasone propionate <500 μg/day or equivalent at baseline.

SUPPLEMENTARY TABLE S2 Baseline demographics and clinical characteristics in patients with and without PAO at baseline by baseline FeNO level

	Patients with PAO		Patients without PAO	
Demographic/characteristic	FeNO <25 ppb (n=324)	FeNO ≥25 ppb (n=453)	FeNO <25 ppb (n=261)	FeNO ≥25 ppb (n=283)
Age, years, mean (SD)	55.1 (11.9)	53.2 (12.8)	45.5 (16.4)	42.9 (18.0)
Female, n (%)	208 (64.2)	265 (58.5)	183 (70.1)	187 (66.1)
Age at asthma onset, years, n (%)				
<18	115 (35.5)	122 (26.9)	95 (36.4)	117 (41.3)
18–40	107 (33.0)	180 (39.7)	95 (36.4)	79 (27.9)
>40	102 (31.5)	151 (33.3)	71 (27.2)	87 (30.7)
Time since asthma diagnosis, years, mean (SD)	25.8 (16.9)	22.4 (16.1)	18.9 (13.9)	16.2 (13.0)
BMI, kg·m^{-2}, mean (SD)	29.4 (6.5)	27.7 (6.2)	29.3 (7.1)	28.0 (7.0)
ICS dose, n (%)[#]				
Medium	93 (28.7)	114 (25.2)	110 (42.1)	85 (30.0)
High	230 (71.0)	339 (74.8)	151 (57.9)	198 (70.0)
OCS use, n (%)	28 (8.6)	57 (12.6)	12 (4.6)	25 (8.8)
Number of exacerbations in the past 12 months, per patient				

Mean (SD)	2.7 (1.5)	2.9 (1.5)	2.5 (1.1)	2.6 (1.1)
Median (min, max)	2 (1, 15)	2 (1, 13)	2 (1, 10)	2 (1, 8)
Pre-bronchodilator FEV_1, L, mean (SD)	1.52 (0.53)	1.60 (0.55)	2.17 (0.64)	2.29 (0.73)
Post-bronchodilator FEV_1, L, mean (SD)	1.75 (0.59)	1.86 (0.63)	2.46 (0.68)	2.62 (0.78)
Percent predicted pre-bronchodilator FEV_1, mean (SD)	54.06 (14.65)	54.15 (13.83)	72.26 (14.14)	74.42 (14.60)
Pre-bronchodilator FVC, L, mean (SD)	2.77 (0.90)	2.95 (0.94)	2.91 (0.86)	3.13 (0.92)
Post-bronchodilator FVC, L, mean (SD)	3.05 (0.94)	3.25 (0.99)	3.10 (0.84)	3.34 (0.95)
Pre-bronchodilator FEV_1/FVC %, mean (SD)	55.38 (9.74)	54.48 (8.85)	74.91 (8.49)	73.36 (8.20)
Post-bronchodilator FEV_1/FVC %, mean (SD)	57.60 (9.58)	57.40 (8.78)	79.29 (6.81)	78.60 (6.74)
FEV_1 reversibility, L, mean (SD)	68.0 (168.2)	62.5 (184.5)	121.4 (298.9)	79.0 (264.4)
FEV_1 reversibility, %, mean (SD)	16.12 (16.38)	17.79 (16.18)	15.05 (17.47)	15.95 (15.91)
Serum total IgE, IU/mL, median (min, max)	152 (2, 12 823)	207 (2, 11 860)	123 (2, 5995)	258 (2, 9741)

BEC, cells·μL^{-1}, median (IQR)	220 (130–385)	320 (200–560)	170 (100–280)	300 (170–490)
FeNO, ppb, median (min, max)	14.0 (4.0, 24.5)	48.0 (25.0, 276.3)	14.0 (3.5, 24.0)	53.0 (25.0, 265.0)

BEC: blood eosinophil count; BMI: body mass index; FeNO: fractional exhaled nitric oxide; FEV_1: forced expiratory volume in 1 second; FVC: forced vital capacity; ICS: inhaled corticosteroid; IgE: immunoglobulin E; IQR: interquartile range; OCS: oral corticosteroid; PAO: persistent airflow obstruction; SD: standard deviation.

[#]Medium-dose ICS: fluticasone propionate 250–500 μg/day or equivalent; high-dose ICS: fluticasone propionate >500 μg/day or equivalent. There was one patient in NAVIGATOR with PAO and a FeNO level <25 ppb (placebo group) who received fluticasone propionate <500 μg/day or equivalent.

SUPPLEMENTARY TABLE S3 Sensitivity analyses in patients with and without PAO at screening/run-in and baseline

	Tezepelumab 210 mg Q4W			Placebo			Tezepelumab 210 mg Q4W vs placebo
**Change in pre-bronchodilator FEV_1 from baseline to week 52, L**							
	Baseline, mean (SD)	n	LS mean (SE) change from baseline	Baseline, mean (SD)	n	LS mean (SE) change from baseline	LS mean difference (95% CI)
**Patients with PAO at screening/run-in and baseline**	1.52 (0.52)	333	0.23 (0.023)	1.59 (0.56)	335	0.05 (0.023)	0.17 (0.11, 0.24)
**Patients without PAO at screening/run-in and baseline**	2.18 (0.70)	288	0.22 (0.025)	2.15 (0.69)	293	0.13 (0.025)	0.09 (0.03, 0.16)
**Change in post-bronchodilator FEV_1 from baseline to week 52, L**							
	Baseline, mean (SD)	n	LS mean (SE) change from baseline	Baseline, mean (SD)	n	LS mean (SE) change from baseline	LS mean difference (95% CI)
**Patients with PAO at screening/run-in and baseline**	1.76 (0.59)	307	0.17 (0.022)	1.84 (0.64)	300	0.01 (0.022)	0.16 (0.10, 0.21)
**Patients without PAO at**	2.48 (0.77)	259	0.10 (0.024)	2.47 (0.73)	259	0.02 (0.024)	0.09 (0.02, 0.15)

**screening/run-in and baseline**					
**AAER over 52 weeks**					
	n	Rate (95% CI)	n	Rate (95% CI)	Rate ratio (95% CI)
**Patients with PAO at screening/run-in and baseline**	336	0.82 (0.67, 0.99)	336	2.18 (1.83, 2.58)	0.38 (0.29, 0.48)
**Patients without PAO at screening/run-in and baseline**	290	0.70 (0.56, 0.87)	294	1.61 (1.33, 1.94)	0.43 (0.33, 0.57)
**Proportion of patients with PAO at screening/run-in and baseline who no longer had PAO at week 52**					
		Proportion of patients who transitioned, % (n/N)		Proportion of patients who transitioned, % (n/N)	Odds ratio (95% CI)
		8.8 (50/566)		4.7 (26/559)	2.00 (1.22, 3.26)

AAER: annualized asthma exacerbation rate; CI: confidence interval; FEV_1: forced expiratory volume in 1 second; LS: least-squares; PAO: persistent airflow obstruction; Q4W: every 4 weeks; SD: standard deviation; SE: standard error.

A total of 672 patients (tezepelumab, n=336; placebo, n=336) had PAO at screening/run-in and baseline and 584 did not (tezepelumab, n=290; placebo, n=294); 78 patients (tezepelumab, n=39; placebo, n=39) had missing data at screening/run-in.

SUPPLEMENTARY TABLE S4 Baseline demographics and clinical characteristics of placebo recipients grouped by whether they had PAO at baseline and/or week 52

Demographic/characteristic	Patients with PAO at baseline and at week 52 (n=309)	Patients without PAO at baseline or at week 52 (n=200)	Patients without PAO at baseline and with PAO at week 52 (n=42)	Patients with PAO at baseline and without PAO at week 52 (n=39)
Age, years, mean (SD)	54.7 (11.5)	42.7 (17.0)	51.4 (15.5)	46.8 (13.4)
Female, n (%)	181 (58.6)	150 (75.0)	22 (52.4)	27 (69.2)
Time since asthma diagnosis, years, mean (SD)	23.4 (16.2)	17.3 (12.9)	20.6 (16.1)	21.2 (14.5)
BMI, $kg \cdot m^{-2}$, mean (SD)	28.2 (6.1)	28.7 (7.5)	27.9 (7.0)	29.04 (6.99)
ICS dose, n (%)[#]				
Medium	96 (31.1)	74 (37.0)	13 (31.0)	10 (25.6)
High	212 (68.6)	126 (63.0)	29 (69.0)	29 (74.4)
OCS use, n (%)	32 (10.4)	12 (6.0)	3 (7.1)	1 (2.6)
Number of exacerbations in the past 12 months, per patient				
Mean (SD)	2.8 (1.5)	2.4 (0.9)	2.5 (1.1)	2.7 (1.3)
Median (min, max)	2.0 (1, 11)	2.0 (1, 6)	2.0 (1, 8)	2.0 (1, 8)
Pre-bronchodilator FEV_1, L, mean (SD)	1.58 (0.54)	2.25 (0.69)	2.13 (0.67)	1.69 (0.60)

Post-bronchodilator FEV_1, L, mean (SD)	1.84 (0.62)	2.58 (0.72)	2.41 (0.73)	1.99 (0.75)
Percent predicted pre-bronchodilator FEV_1, mean (SD)	54.68 (14.24)	74.06 (14.73)	70.29 (11.60)	56.22 (11.38)
Pre-bronchodilator FVC, L, mean (SD)	2.93 (0.96)	3.00 (0.84)	3.14 (1.00)	2.76 (1.01)
Post-bronchodilator FVC, L, mean (SD)	3.23 (1.02)	3.23 (0.86)	3.24 (0.93)	3.06 (1.11)
Pre-bronchodilator FEV_1/FVC %, mean (SD)	54.46 (8.31)	75.18 (8.16)	68.52 (7.34)	61.99 (7.68)
Post-bronchodilator FEV_1/FVC %, mean (SD)	57.05 (8.37)	79.79 (6.92)	74.06 (5.07)	64.91 (4.90)
Reversibility in FEV_1, L, mean (SD)	71.99 (193.91)	120.65 (309.16)	24.15 (83.53)	119.36 (259.37)
FEV_1 percent reversibility, n (%)				
<12	136 (44.0)	100 (50.0)	24 (57.1)	15 (38.5)
≥12 and <15	29 (9.4)	14 (7.0)	5 (11.9)	6 (15.4)
≥15	144 (46.6)	86 (43.0)	13 (31.0)	18 (46.2)
Serum total IgE, IU/mL, median (min, max)	161 (2, 11 860)	179 (2, 9741)	231 (3, 2337)	195 (6, 7406)
FEIA positive for any perennial aeroallergen, n (%)¶	177 (57.3)	127 (63.5)	26 (61.9)	27 (69.2)

BEC, cells·μL^{-1}, median (IQR)	280 (150–460)	240 (130–400)	180 (130–340)	360 (140–690)
FeNO, ppb, median (min, max)	29.0 (5.0, 276.3)	24.0 (3.5, 231.0)	31.5 (5.0, 176.0)	32.0 (6.0, 258.0)

BEC: blood eosinophil count; BMI: body mass index; FEIA: fluorescence enzyme immunoassay; FeNO: fractional exhaled nitric oxide; FEV_1: forced expiratory volume in 1 second; FVC: forced vital capacity; ICS: inhaled corticosteroid; IgE: immunoglobulin E; IQR: interquartile range; OCS: oral corticosteroid; PAO: persistent airflow obstruction; SD: standard deviation.

[#]Medium-dose ICS: fluticasone propionate 250–500 μg/day or equivalent; high-dose ICS: fluticasone propionate >500 μg/day or equivalent. There was one patient in NAVIGATOR with PAO (placebo group) who received fluticasone propionate <500 μg/day or equivalent. [¶]Positive for at least one perennial aeroallergen (cat dander, dog danger, cockroach, dust mite [*Dermatophagoides farinae* or *D. pteronyssinus*] and mould mix).
